# Network analysis of live pig movements in North Macedonia: Pathways for disease spread

**DOI:** 10.3389/fvets.2022.922412

**Published:** 2022-08-09

**Authors:** Kathleen C. O'Hara, Daniel Beltrán-Alcrudo, Mark Hovari, Blagojcho Tabakovski, Beatriz Martínez-López

**Affiliations:** ^1^Center for Animal Disease Modeling and Surveillance (CADMS), School of Veterinary Medicine, University of California, Davis, Davis, CA, United States; ^2^Food and Agriculture Organization of the United Nations (FAO), Regional Office for Europe and Central Asia, Budapest, Hungary; ^3^Food and Veterinary Agency, Republic of North Macedonia, Skopje, North Macedonia

**Keywords:** African swine fever, North Macedonia, social network analysis, transboundary animal disease, *Sus scrofa*

## Abstract

Globalization of trade, and the interconnectivity of animal production systems, continues to challenge efforts to control disease. A better understanding of trade networks supports development of more effective strategies for mitigation for transboundary diseases like African swine fever (ASF), classical swine fever (CSF), and foot-and-mouth disease (FMD). North Macedonia, bordered to the north and east by countries with ongoing ASF outbreaks, recently reported its first incursion of ASF. This study aimed to describe the distribution of pigs and pig farms in North Macedonia, and to characterize the live pig movement network. Network analyses on movement data from 2017 to 2019 were performed for each year separately, and consistently described weakly connected components with a few primary hubs that most nodes shipped to. In 2019, the network demonstrated a marked decrease in betweenness and increase in communities. Most shipments occurred within 50 km, with movements <6 km being the most common (22.5%). Nodes with the highest indegree and outdegree were consistent across years, despite a large turnover among smallholder farms. Movements to slaughterhouses predominated (85.6%), with movements between farms (5.4%) and movements to market (5.8%) playing a lesser role. This description of North Macedonia's live pig movement network should enable implementation of more efficient and cost-effective mitigation efforts strategies in country, and inform targeted educational outreach, and provide data for future disease modeling, in the region.

## Introduction

The ongoing globalization of agricultural trade has increased the international movement of animals, animal products, and disease (1–3). African swine fever (ASF), classical swine fever (CSF), and foot-and-mouth disease (FMD) are all examples of transboundary animal diseases (TADs), whose introduction would be devastating to local economies, food supply chains and animal production industries (4). ASF, in particular, has been of growing global concern. This reportable, viral, hemorrhagic disease of pigs, re-emerged from Africa into Georgia in 2007 and has steadily spread westward through Europe, northeastward across Russia into China in 2018, and most recently to Hispaniola in the Caribbean in 2021 (5). The ongoing spread and associated risk of severe economic losses if ASF is introduced, have provided the impetus for many countries to develop and enhance their surveillance and response planning.

The Republic of North Macedonia is located on the Balkan peninsula in Southeastern Europe, bordered by Kosovo and Serbia to the North, Bulgaria to the East, Greece to the South, and Albania to the West. While CSF and FMD have been well-controlled in the Balkans through recent years, ASF continues to spread. Ongoing ASF outbreaks have been reported in both domestic pigs and wild boar in Bulgaria and Serbia, while Greece reported and was able to control a single introduction into domestic pigs in 2020 (5). North Macedonia reported its first cases of ASF in domestic pigs in January 2022, and in wild boar in March 2022 (5). This introduction has increased the need for traceability and efficient data-driven methods to support disease surveillance, prevention, and outbreak response. The Food and Agriculture Organization of the United Nations (FAO) recently completed a survey in North Macedonia describing their pig sector (6), and continues to work toward enhancing and implementing targeted surveillance.

This work aims to describe North Macedonia's pig census and the live pig movement network to support ongoing disease mitigation planning and response efforts. Understanding the social network of this sector will support risk assessment of disease dissemination within the local industry (7), as well as faster responses to new detections (8). ASF transmission in both domestic and wild pigs can occur *via* direct contact with an infected animal, through consumption of contaminated materials (e.g., swill feeding, discarded offal, scavenged carcasses or garbage), exposure to fomites, iatrogenically, or through the bite of infected *Ornithodoros* ticks if present in the area (9–15). Therefore, movement of infected live pigs, pork products, or contaminated fomites, provides opportunities for disease introduction and spread. Understanding when, where and how frequently these contacts occur, and the network structure and vulnerabilities, may help to strategically allocate risk-based, more cost-effective, preventive and control measures.

Social network analysis (SNA) has been demonstrated to be a valuable tool to describe pig movement network structures and has been used with increasing frequency in the swine industry (16–31). It has been used to evaluate the movement network dynamics and helps to quickly identify the individual farms, areas and time periods that may pose the highest risk for disease introduction to the system (32–34). These insights allow for implementation of risk mitigation strategies at these spatial or temporal hotspots (34), as well as more realistic disease modeling.

Understanding the network of pig movements in North Macedonia is the first step toward risk analysis. Currently, there is very limited published information about the pig sector in North Macedonia and the Balkans. This lack of information is a critical gap in animal health and outbreak response planning. The predominance of small-scale subsistence farmers in North Macedonia, highlights the potential impact of a TAD of swine on food production and security in the country (6). SNA applied to the pig industry may also allow for the identification of potential super-spreaders (nodes likely to spread disease fastest or to the most additional nodes given their network contacts) or super-receivers (nodes at highest risk of disease exposure due to receipt of incoming movements from the most other nodes) of disease within North Macedonia's pork industry chain, providing targeted locations for increased surveillance and risk mitigation (33, 35).

This study aimed to provide one of the first descriptions of North Macedonia's pig population and the social network of its live pig trade. Our primary objectives were: to describe the distribution of pigs and pig farms; to describe the live pig network structure; to describe pig movement spatio-temporal dynamics; and to identify priority farms that may contribute to the risk of disease introduction and spread. An increased availability of pig demographic and movement data in North Macedonia and the Balkan region will help to better understand, and even predict, disease transmission patterns, supporting risk-based surveillance and control strategies for both endemic and emerging pig diseases such as ASF.

## Materials and methods

### Data

Annual pig census data for 2016–2020 was provided by the Veterinary Authority (Food and Veterinary Agency of North Macedonia). The 2016–2017 census data provided unique identification number (UIN), town/village, region, and number of animals, for each farm. Census data for 2018–2020 included UIN, coordinates, town/village, region, total number of animals, number of piglets (pigs <25 kg), number of fattening pigs, number of gilts, number of sows, and number of boars. North Macedonia is divided into increasingly smaller administrative levels from regions, to municipality, to towns/villages.

Records of permitted movements of live pigs for 2017–2019 were provided by the Veterinary Authority. Permits are required for movement between farms, markets and slaughterhouses. Movement records for 2017–2018 provided data for the entire year, while 2019 data covered only January 1 to November 23, 2019. Records for 2017–2018 included movement type (completed movement, departed, departed with no document, movement off holding, movement without document), certificate number, date of departure, date of arrival, number of pigs departing, number of pigs arriving, origin UIN, destination UIN, and type of UIN for both origin and destination (farm, market, slaughterhouse, or unspecified). North Macedonian markets are registered live animal markets, that include a variety of species. Records for 2019 included movement type, date of departure, date of arrival, for origin and destination: UIN, town/village, municipality, region, herd type, and coordinates were provided. Number of animals moved was not provided for 2019. A separate set of data on movements to slaughterhouses was provided for 2019, which included date of departure, date of arrival, origin and destination: UIN, town/village, municipality, region, number of animals departed, number of animals arrived. Therefore, the number of animals moved is only available for movements to slaughterhouses for 2019.

UIN types that were unspecified were assumed to be farms: 664 (3.0%) origin types and 304 (1.4%) destination types were reassigned from unspecified to farm. All of the 13 commercial slaughterhouses in North Macedonia were identified (no slaughterhouses were mis-identified as a farm or market). Five hundred fifty-nine (2.6%) movements did not have a destination recorded and were not considered in the network analysis. A total of 21,801 movements were included in this study.

Coordinate information for 2017–2018 movements were referenced from 2019 movement and census data by UIN. The remaining unassigned UIN's were assigned to the town/village centroid using the UIN coding system in which the first four digits reference a specific town/village. In 2017, this represented 53 (12.8%) UINs, associated with 127 (1.8%) of movements. In 2018, this represented 45 (10.9%) UINs, accounting for 110 (1.4%) of movements.

Data were collected, validated and cleaned in Microsoft Excel 2016 and R Studio (v.3.6.1) (36, 37).

### Census

Descriptive statistics were calculated for the census data in R Studio, excluding farms that reported zero total animals. Spatial visualization and analyses were performed in ArcGIS Desktop v10.7. Mapping was conducted using the World Azimuthal Equidistant Projection.

### Network analysis

The UINs of premises in the census, and reporting movements each year, were highly variable, with only 618 (23.2%) being reported across all census years (Supplementary Figure S1) and only 163 (34.0%) present in the movement records across all years. In general, larger commercial farms were more stable year to year, while the UINs of smaller backyard farms were not consistently present in the census across time. The movement networks were therefore analyzed separately for each month and year. Static, directed, unweighted networks for were defined using pig production sites as nodes or vertices, and shipments of live pigs as edges. The similarity between annual networks was calculated using a Jaccard index, determining the proportion of nodes and edges that were shared between networks (38). Jaccard similarity was calculated using the gasub package (39) in R Studio (v 3.6.1) (37). The properties and characteristics of the networks for each month and year were described using network parameters including number of nodes, number of edges, diameter, edge density, average path length, and transitivity. Centrality measures of in-degree and out-degree were calculated for each node. In-degree is defined as the number of incoming shipments to a production site, out-degree is the number of outgoing shipments from a production site (35, 40, 41). Betweenness is the number of shortest paths between any pair of nodes in the network that pass through an individual node (40–42). Diameter is the longest of all the shortest path lengths between nodes in the network (35, 40, 41). Edge density is the ratio of the number of edges observed in the network to the number of possible edges (35, 40, 41). Average path length is the mean length of all the shortest paths between nodes in the network (43). Transitivity coefficient is the proportion of nodes inter-connected by a single intermediate node that are also themselves connected; this parameter is also known as the clustering coefficient (41, 43). The igraph package (v 1.1.2) (44) was used to generate and describe the static networks and evaluate network parameters. Edge density, diameter, average path length and transitivity were calculated under the igraph package using functions: edge_density, diameter, mean_distance, and transitivity, respectively. Type global was used for the transitivity function. The degree distribution of each annual network was evaluated using the networkProperties function from the splineTimeR package (45).

Components are subregions within a network in which all nodes are directly or indirectly linked. For directed networks, components can be classified as strong or weak. Strong components are those in which every node can reach every other node by connected paths, while weak components are areas in which every node is connected when we ignore directionality (25, 46). The giant weak component (GWC) is the largest weak component, and the giant strong component (GSC) is the largest strong component (46). GWC and GSC were assessed for each network using the components function within igraph (44). The Walktrap community finding algorithm was used to define communities within each year's network (47). Default settings were applied to weighted networks using a step-length of 4. Within the walktrap_community function weighted edges have an increased probability of selection by the random walker.

Weighted annual networks were used to evaluate the consistency of nodes shipping and receiving animals. Weighted networks with slaughterhouses removed were used to specifically evaluate nodes involved in the shipping and receiving animals expected “to live” or remain in the value chain. Weighted networks were also used to summarize movement distances. Movement distances were calculated based on Veness's equation for Excel derived from the spherical law of cosines (48, 49).

### Mapping movements

To address the large turnover in UINs each year, annual movements were also summarized at the municipality level. For each year, the number of movements into and out of each municipality was calculated and mapped for visual comparison. Summary data was mapped in ArcGIS for visual analysis.

## Results

### Census

Census data were summarized in Table 1 and Supplementary Table S1. In general, the reported number of farms and number of pigs in North Macedonia have been increasing. The highest number of farms were reported in 2018 and 2020. The total number of farms increased by 31.6% between 2016 and 2020, while the number of pigs increased by 17.7%. Smallholder farms ( ≤ 10 pigs) saw the largest expansion. Consistent with this, the median and average number of pigs per farm decreased during this period. In the most recent data from 2020, there is a median of 3 pigs per farm. The Vardar (288.7%) and Eastern (260.9%) regions had the largest proportional increase in number of farms; the Northeastern (20.6%) and Polog (16.9%) regions had decreases in the number of farms. Skopje (46.6%) and the Southwestern (41.2%) regions had the largest proportional increase in number of pigs. Despite the decrease in number of farms in the Polog region, the number of pigs increased.

**Table 1:** North Macedonia pig census data 2016–2020 summarized on the country and regional level.

		**2016**	**2017**	**2018**	**2019**	**2020**
**Country**						
Number of farms	Total	1,976	2,344	3,096	2,175	2,889
	With ≤ 10 pigs	1,262 (63.9%)	1,619 (69.1%)	2,589 (83.6%)	1,664 (76.5%)	2,339 (81.0%)
	With 11–100 pigs	638 (32.3%)	652 (27.8%)	435 (14.1%)	434 (20.0%)	465 (16.1%)
	With >100 pigs	76 (3.8%)	73 (3.1%)	72 (2.3%)	77 (3.5%)	85 (2.9%)
Number of pigs	Total	109,845	110,058	128,983	125,230	133,448
	Avg per farm	55.6	47.0	41.7	57.6	46.2
	Median per farm	6	4	3	4	3
	Range (Min-Max)	1–18,576	1–19,837	1–21,747	1–22,459	1–21,159
**Region**						
Eastern	Number farms	215	258	626	194	776
	Number pigs	31,075	31,520	34,504	33,593	40,703
Northeastern	Number farms	927	1,205	918	874	736
	Number pigs	12,592	11,977	10,602	10,519	10,454
Pelagonia	Number farms	35	50	196	170	63
	Number pigs	1,034	1,169	1,606	1,867	1,146
Polog	Number farms	237	262	344	274	197
	Number pigs	13,982	14,249	16,663	14,923	16,000
Skopje	Number farms	15	14	19	19	28
	Number pigs	1,653	1,951	1,760	1,751	2,424
Southeastern	Number farms	289	242	371	165	480
	Number pigs	8,177	7,034	8,275	8,149	7,568
Southwestern	Number farms	196	230	418	344	368
	Number pigs	1,652	1,813	2,720	1,959	2,333
Vardar	Number farms	62	83	204	135	241
	Number pigs	39,680	40,345	52,853	52,469	52,820

**Figure 1:**
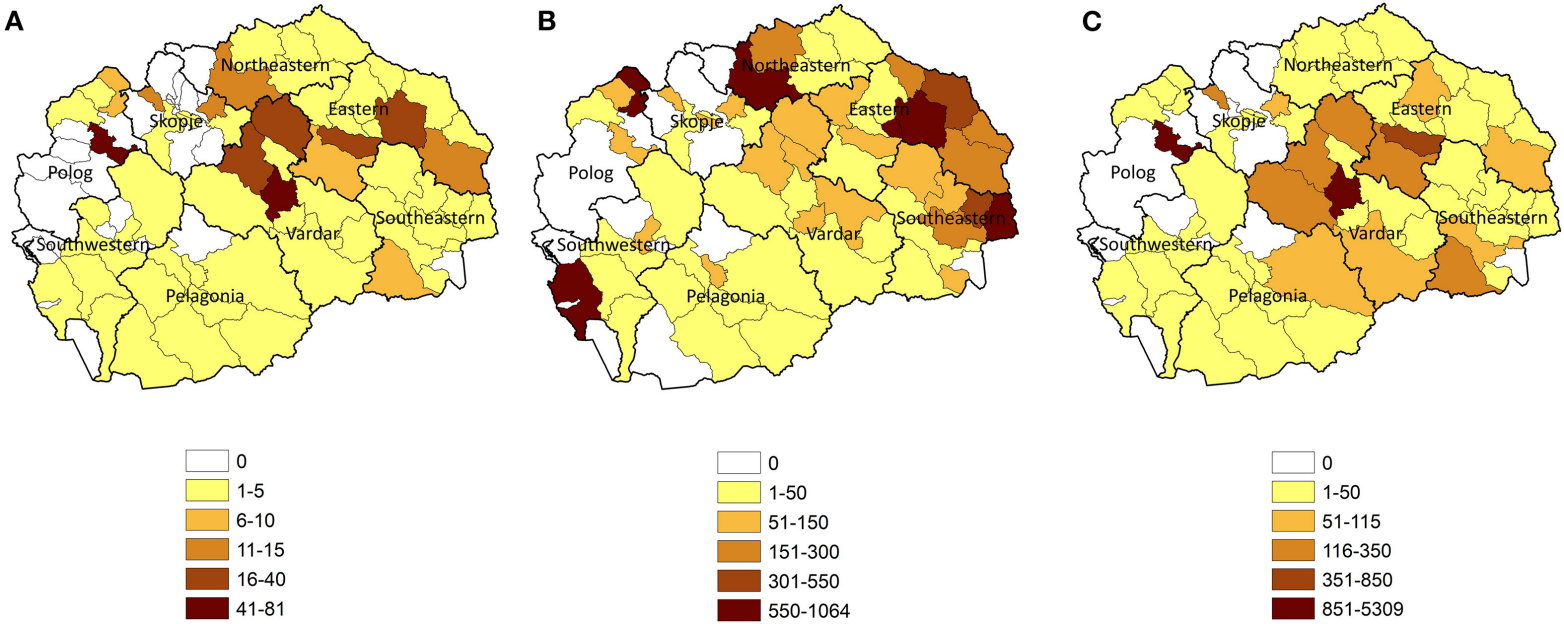
North Macedonian 2020 pig census summarized at the municipality level by **(A)** pig per square kilometer, **(B)** farms per 1,000 square kilometers, and **(C)** pigs by farm. Black lines outline regions. Gray lines outline municipalities.

Based on the 2020 census, the highest densities of pigs are present in the Vardar, Polog and Eastern regions, while the highest densities of farms are in the Northeastern, Eastern and Southeastern regions (Figure 1). Vardar has the highest number of pigs per farm. Smallholder farms are distributed throughout the country.

### Network analysis

**Figure 2:**
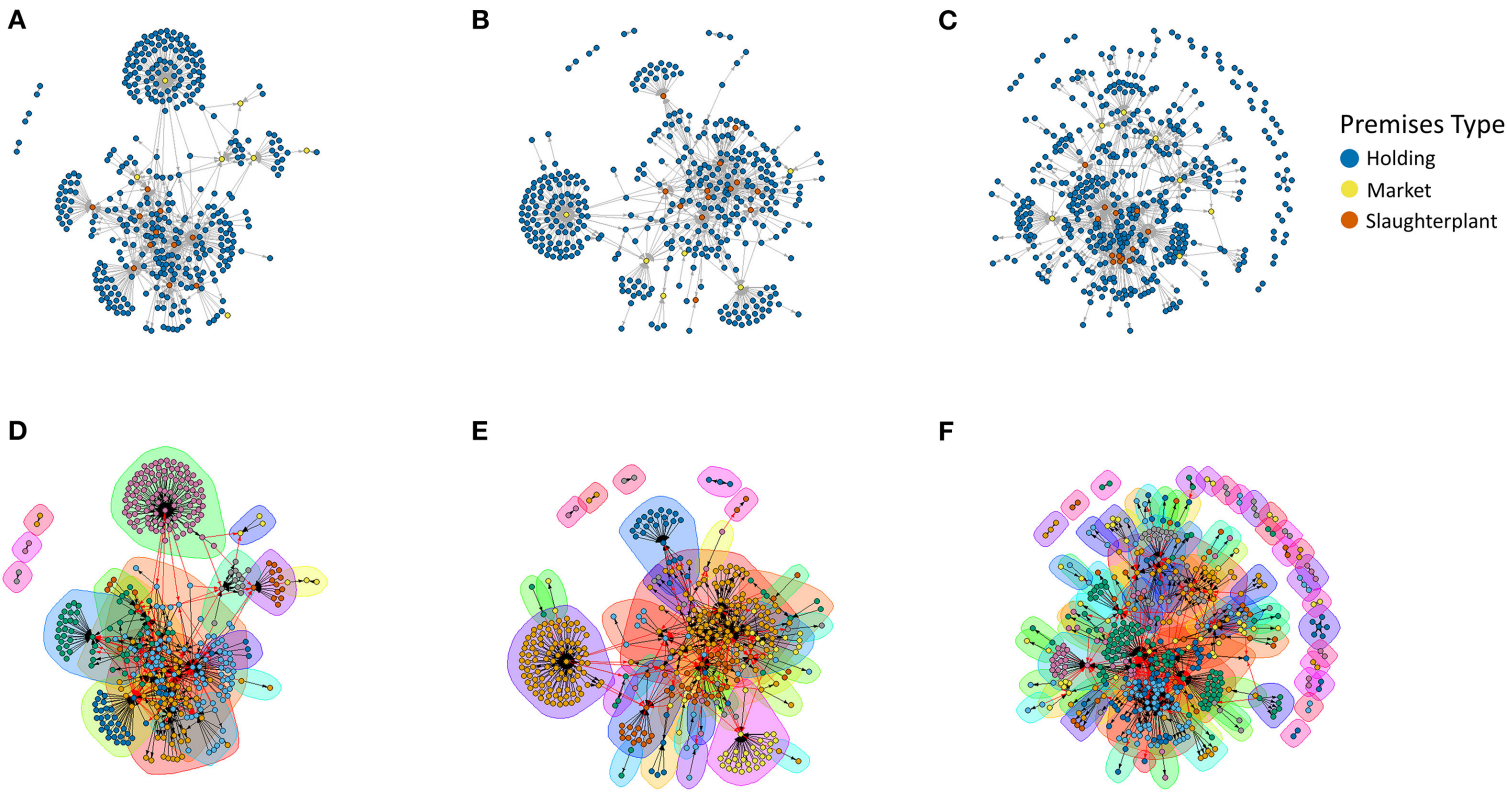
Non-spatially explicit simplified (repeated edges and loops removed) North Macedonian live pig movement networks for **(A)** 2017, **(B)** 2018, and **(C)** 2019. Communities within the network were identified using the Walktrap community finding algorithm, with **(D)** 17 communities identified in 2017, **(E)** 33 communities identified in 2018, and **(F)** 85 communities identified in 2019.

Network parameters for each month and year's static network are presented in Table 2. Generally, network parameters were consistent throughout a given year. Even with incomplete data for 2019, the number of nodes increased by 23.4% between 2017 and 2019, and the number of edges or movements increased by 6.5%. Jaccard similarity for the annual networks showed that 44.9% of nodes were shared between 2017 and 2018, 28.4% between 2017 and 2019, and 28.5% between 2018 and 2019. Looking at shared edges, Jaccard similarity showed 33.0% of edges were shared between 2017 and 2018, 19.5% between 2017 and 2019, and 22.2% between 2018 and 2019. The observed range of indegree was more stable than that of outdegree year to year; the maximum outdegree increased by 32.4% between 2017 and 2018 and 11.3% between 2017 and 2019 (Table 1). The degree distributions were highly right skewed, and generally followed power-law distributions (Supplementary Figure S2). All networks form weakly-connected components, and are characterized by short diameters and path lengths, and low transitivity. A marked decrease in betweenness was observed in 2019, compared with 2017-2018. The GSC was composed of 4 nodes in 2017, 5 in 2018 and 23 nodes in 2019. The GWC consistently made up the majority of the network, 89.9% in 2017, 91.5% in 2018, and 100% in 2019. Markets and slaughterhouses are observed to be aggregation points for incoming movements across years (Figures 2A–C). The 2019 network has more frequent occurrences of movements between two nodes that are otherwise independent of the rest of the network (Figure 2C). Community identification algorithms identified 17 communities in 2017 (nodes in community: mean: 23, median: 9, range 2–106), 33 in 2018 (nodes in community: mean: 12, median: 2, range: 2–100), and 85 in 2019 (nodes in community: mean: 6, median: 2, range 2–106: 1–75). An increase in nodes that only contact one other node in the network was observed in 2018–2019 (Figures 2D–F). When evaluated spatially, across all years, while some communities remain highly localized, there are communities that bridge regions and, in some cases, span the entire country. When evaluating a simplified network, removing repeated shipments, the median distance of a shipments across all years was 28.9 kilometers (average: 41.0, range: 0–187.5; note zero values reflect movements in which nodes were assigned to the same town centroid). The distribution of shipment distances (km) was stable between 2017 (median: 27.2, average: 38.2, range: 0–184.3) and 2018 (median: 25.7, average: 35.5, range: 0–176.8), with a moderate increase observed in 2019 (median: 41.8, average: 48.9, range: 0–187.5). When evaluating all shipments across all years, most shipments occur over distances <50 km; each year the largest number of shipments occurred within 0–6 kilometers (Supplementary Figure S3).

**Table 2:** North Macedonia live pig movement network parameters by month and year for 2017–2019.

	**Jan**	**Feb**	**Mar**	**Apr**	**May**	**Jun**	**Jul**	**Aug**	**Sep**	**Oct**	**Nov**	**Dec**	**Year**
**2017**													
Nodes	121	141	160	148	143	125	124	126	128	124	133	157	388
Edges	506	511	584	581	576	482	550	538	507	561	600	682	6,678
Indegree [Mean (Min, Max)]	4.18 (0, 120)	3.62 (0, 123)	3.65 (0, 130)	3.93 (0, 135)	4.03 (0, 131)	3.86 (0, 111)	4.44 (0, 105)	4.27 (0, 107)	3.96 (0, 108)	4.52 (0, 127)	4.51 (0, 132)	4.34 (0, 140)	17.21 (0, 1,469)
Outdegree [Mean (Min, Max)]	4.18 (0, 49)	3.62 (0, 43)	3.65 (0, 45)	3.93 (0, 50)	4.03 (0, 54)	3.86 (0, 50)	4.44 (0, 53)	4.27 (0, 55)	3.96 (0, 52)	4.52 (0, 49)	4.51 (0, 54)	4.34 (0, 63)	17.21 (0, 584)
Betweenness [Mean (Min, Max)]	0.03 (0, 2)	0.65 (0, 84)	2.11 (0, 234)	1.15 (0, 138)	0.07 (0, 4)	0.07 (0, 2)	0.06 (0, 3)	0.37 (0, 39)	0.39 (0, 42)	0.06 (0, 4)	0.73 (0, 91)	2.31 (0, 265)	26.01 (0, 5,922)
Edge density	0.03	0.03	0.02	0.03	0.03	0.03	0.04	0.03	0.03	0.04	0.03	0.03	0.04
Diameter	2	2	3	3	2	2	2	2	2	2	2	4	5
Average path length	1.03	1.3	1.67	1.49	1.05	1.06	1.05	1.24	1.24	1.04	1.37	1.75	2.47
Transitivity	0.017	0.011	0.009	0.013	0.016	0.008	0.018	0.011	0.012	0.019	0.016	0.011	0.014
GSC^*^ size	2	2	3	6	4	5	7	4	1	3	2	3	4
GWC^*^ size	121	137	143	139	141	125	124	123	126	124	128	151	349
Communities	12	14	12	17	13	14	13	15	13	15	10	17	17
**2018**													
Nodes	128	142	149	156	135	123	127	120	124	139	149	166	387
Edges	560	523	558	645	591	527	657	593	558	710	694	835	7,451
Indegree [Mean (Min, Max)]	4.38 (0, 118)	3.68 (0, 108)	3.75 (0, 114)	4.14 (0, 131)	4.38 (0, 119)	4.29 (0, 103)	5.17 (0, 123)	4.94 (0, 94)	4.50 (0, 93)	5.11 (0, 121)	4.66 (0, 142)	5.03 (0, 143)	19.25 (0, 1,404)
Outdegree [Mean (Min, Max)]	4.38 (0, 52)	3.68 (0, 47)	3.75 (0, 54)	4.14 (0, 73)	4.38 (0, 75)	4.29 (0, 62)	5.17 (0, 71)	4.94 (0, 64)	4.50 (0, 68)	5.11 (0, 76)	4.66 (0, 68)	5.03 (0, 89)	19.25 (0, 773)
Betweenness [Mean (Min, Max)]	1.21 (0, 60)	0.74 (0, 78)	1.05 (0, 108)	0.05 (0, 2)	0.50 (0, 42)	0.93 (0, 92)	1.31 (0, 114)	1.08 (0, 105)	0.40 (0, 37)	1.55 (0, 205)	1.01 (0, 127)	1.04 (0, 143)	15.68 (0, 4,682)
Edge density	0.03	0.03	0.03	0.03	0.03	0.04	0.04	0.04	0.04	0.04	0.03	0.03	0.05
Diameter	4	3	3	2	3	3	3	3	3	2	3	3	5
Average path length	1.63	1.4	1.52	1.04	1.31	1.45	1.61	1.48	1.25	1.56	1.46	1.48	2.12
Transitivity	0.02	0.019	0.013	0.013	0.018	0.022	0.014	0.024	0.03	0.012	0.011	0.015	0.023
GSC^*^ size	3	3	6	6	2	6	4	3	3	3	2	2	5
GWC^*^ size	125	136	142	156	133	118	124	115	122	134	144	159	354
Communities	16	12	21	22	14	16	14	15	13	14	12	14	33
**2019**													
Nodes	140	176	167	174	165	125	128	148	155	148	136	0	479
Edges	596	655	628	751	669	585	712	642	679	698	498	0	7,113
Indegree [Mean (Min, Max)]	4.26 (0, 128)	3.72 (0, 133)	3.76 (0, 121)	4.32 (0, 160)	4.06 (0, 126)	4.69 (0, 117)	5.56 (0, 139)	4.34 (0, 120)	4.38 (0, 156)	4.72 (0, 166)	3.66 (0, 95)		14.85 (0, 1,461)
Outdegree [Mean (Min, Max)]	4.26 (0, 64)	3.72 (0, 60)	3.76 (0, 53)	4.32 (0, 66)	4.06 (0, 64)	4.69 (0, 58)	5.56 (0, 76)	4.34 (0, 60)	4.36 (0, 69)	4.72 (0, 66)	3.66 (0, 50)		14.85 (0, 650)
Betweenness [Mean (Min, Max)]	0.03 (0, 2)	0.04 (0, 2)	0.03 (0, 2)	0.02 (0, 2)	0.05 (0, 4)	0.06 (0, 4)	0.11 (0, 4)	0.04 (0, 2)	0.08 (0, 4)	0.08 (0, 4)	0.07 (0, 3)		0.21 (0, 15)
Edge density	0.03	0.02	0.02	0.02	0.02	0.04	0.04	0.03	0.02	0.03	0.03		0.03
Diameter	2	2	2	2	3	2	3	2	2	2	3		4
Average path length	1.02	1.04	1.03	1.02	1.04	1.05	1.09	1.04	1.07	1.05	1.06		1.13
Transitivity	0.012	0.015	0.025	0.009	0.027	0.017	0.023	0.015	0.015	0.019	0.018		0.014
GSC^*^ size	11	15	16	10	16	11	11	13	12	9	16		23
GWC^*^ size	140	176	167	174	165	125	128	148	155	148	136		479
Communities	19	26	32	27	33	17	25	29	24	22	28		85

* *GSC, giant strongly connected component; GWC, giant weakly connected component*.

**Figure 3:**
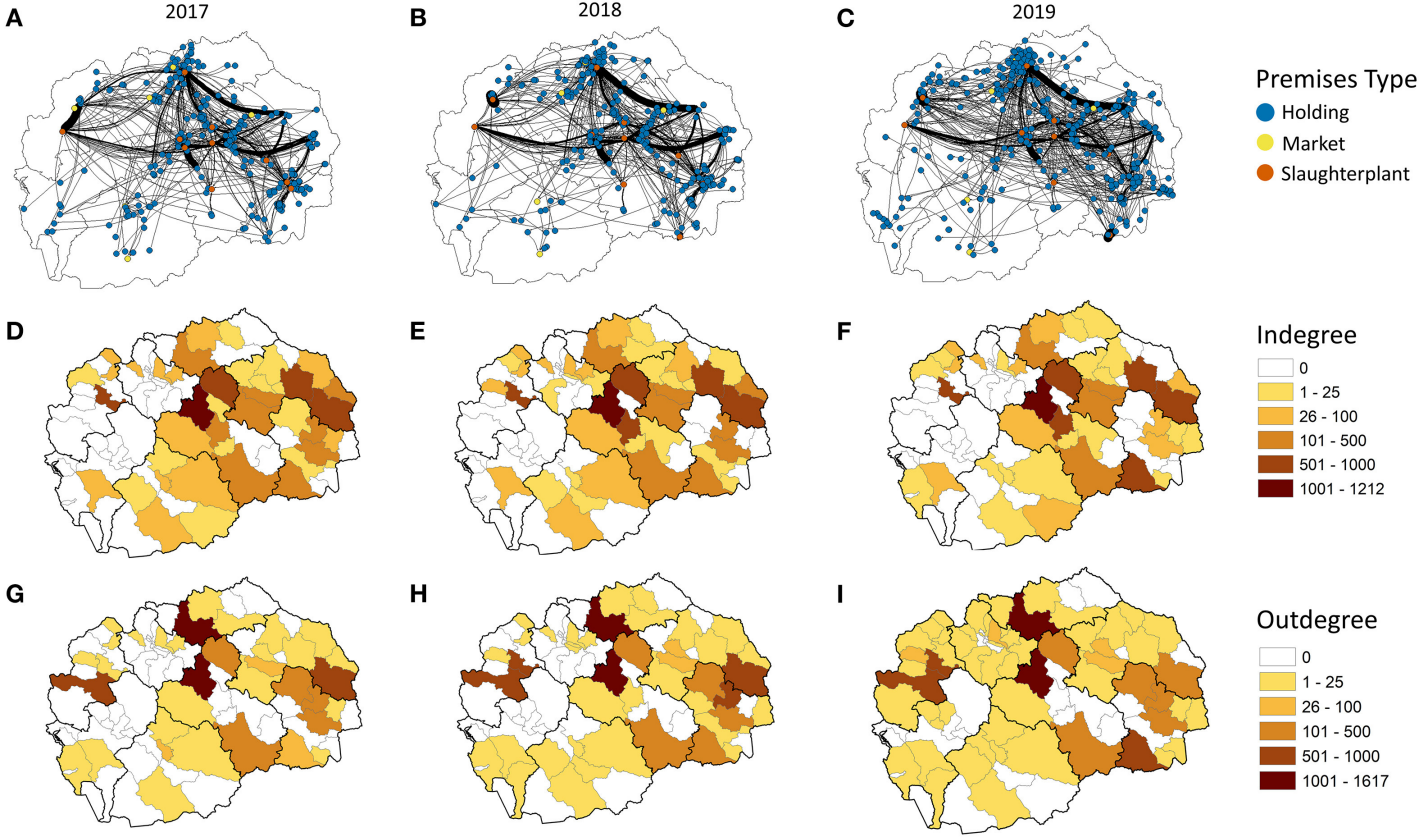
Network analysis of live pig movements in North Macedonia for 2017–2019. Simplified network with edge weight as the number of movements divided by 20 for **(A)** 2017, **(B)** 2018, **(C)** 2019. Summary of the number of live pig movements into a municipality for **(D)** 2017, **(E)** 2018, **(F)** 2019. Summary of the number of live pig movements out of a municipality for **(G)** 2017, **(H)** 2018, **(I)** 2019.

**Figure 4:**
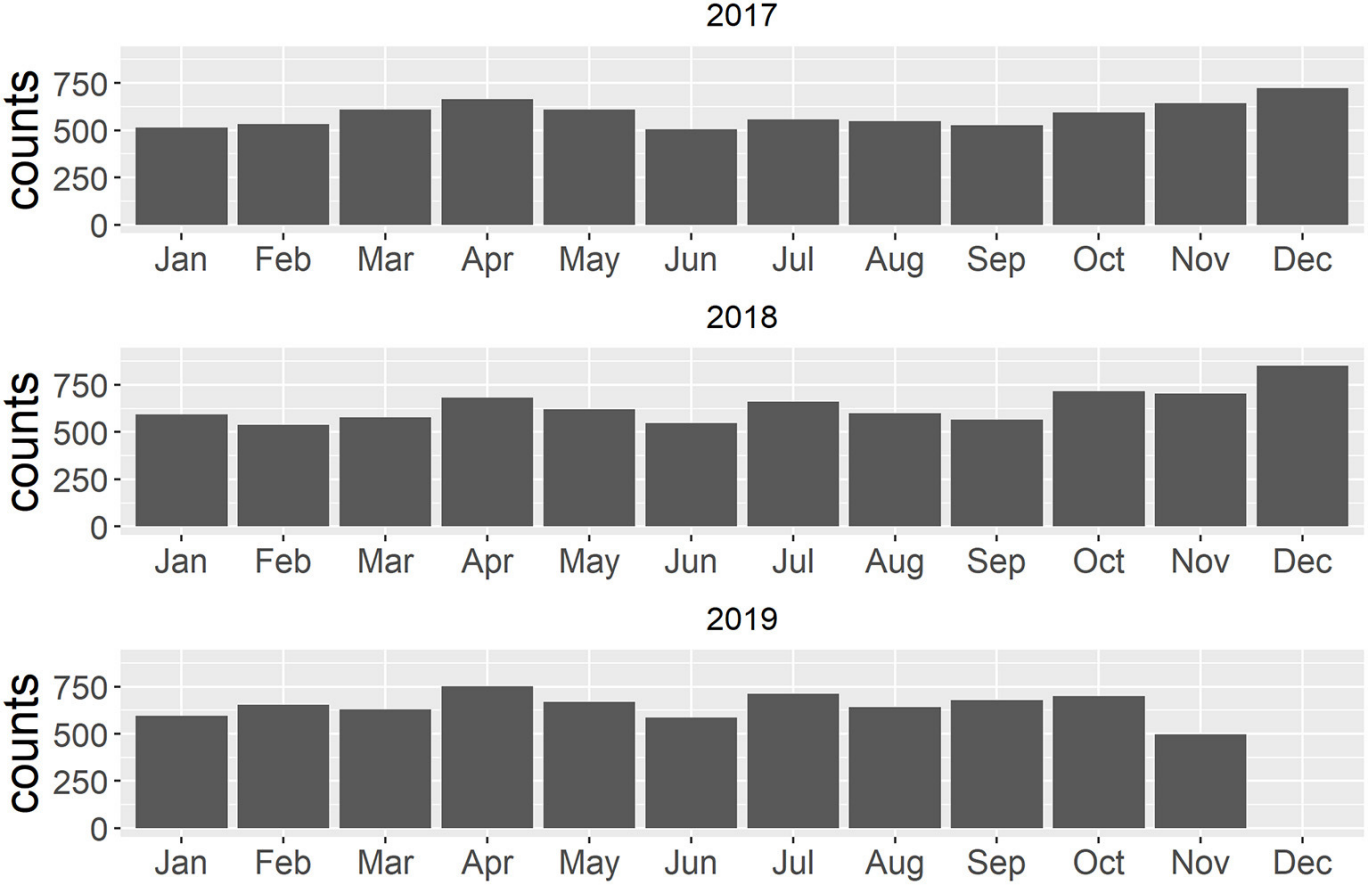
Number of live pig movements within North Macedonia by shipment month for 2017–2019.

When the networks are visualized geospatially, the most frequent and stable movements are those to slaughterhouses (Figure 3). Between 2017 and 2019, 85.6% of movements were to slaughterhouses. A shift in slaughterhouse usage can be observed in Polog between 2017 and 2017 (Figures 3A,B), and in the Southeastern region between 2018 and 2019 (Figures 3B,C). When summarized at the municipality level, the receipt of pigs was spatially consistent across years, with the exception of a municipality in the Southeastern region with a slaughterhouse that received more shipments during years 2018–2019 (Figures 3D–F). Increases in small scale movements out of municipalities can be observed between 2017 and 2019, with increases in the Polog, Skopje and Pelagonia regions most evident (Figures 3G–I). The network demonstrates seasonality, with peaks in the number of movements observed in April, July and November-December (Figure 4). Movements to markets follow this overall trend, with shipments occurring throughout the year, with March-April and November-December peaks. In 2017, 25.3%, and in 2018, 27.5%, of movements to market occurred in November-December; this dropped to 3.9% in 2019.

Those nodes with the highest indegree and highest outdegree are consistent across the years, with 9 of the 10 nodes (all slaughterhouses) with the highest indegree consistent from 2017 to 2019 and likewise 9 of the 10 nodes (all farms) with the highest outdegree consistent from 2017 to 2019. When narrowed to those movements not to slaughterhouses (“to live”), this consistency is largely retained. Among those nodes with the highest indegree in a “to live” network, 7 of the top 10 nodes (4 markets, 3 farms) are consistent between 2017 and 2019. Among those nodes with the highest outdegree in a “to live network” 8 of the top 10 (1 market, 7 farms) nodes are consistent between 2017 and 2018, but that drops to 6 of the top 10 (all farms) in 2019. Receiving and shipping at the highest volumes and throughout the year, these nodes were classified as presumptive super-receivers and super-spreaders using the live pig network as a proxy for disease spread. Summarizing the top 10 nodes for each year together, within a “to live” network, the average indegree per year is 60.5 (median: 42, range: 7–294), while the average outdegree per year is 40.2 (median: 34, range: 14–97).

When evaluating movements by type of origin and destination, the proportions of movements between farms, markets and slaughterhouses are generally consistent between 2017 and 2019 (Table 3). Approximately 5–6% of movements are from one farm to another, about 6% from farm to market, and about 85% from farm to slaughterhouses. Movements from markets compose <2% of movements, with no movements from markets recorded in 2019. The lack of information on destination improved across the years, decreasing from 5.1 to 0.0% of movements between 2017 and 2019 (Table 3).

**Table 3:** Movements of live pigs in North Macedonia from 2017-2020, summarized by type of premises moving from and to.

**Year**	**From**	**To**	**Number**	**Proportion (%)**
2017	Farm	Farm	283	4.0
		Market	384	5.5
		Slaughterhouse	5,949	85.6
		Unknown	352	5.0
	Market	Farm	60	0.9
		Market	0	0
		Slaughterhouse	2	<0.1
		Unknown	5	<0.1
	Total		7,035	
2018	Farm	Farm	375	4.9
		Market	473	6.2
		Slaughterhouse	6,526	85.3
		Unknown	184	2.4
	Market	Farm	77	1.0
		Market	0	0
		Slaughterhouse	0	0
		Unknown	18	<0.1
	Total		7,653	
2019	Farm	Farm	513	7.2
		Market	414	5.8
		Slaughterhouse	6,186	87.0
		Unknown	0	0
	Market	Farm	0	0
		Market	0	0
		Slaughterhouse	0	0
		Unknown	0	0
	Total		7,113	

*Proportion is calculated as the proportion of total movements for a given year*.

Movements between farms were associated with the largest number of pigs, averaging 108.0 pigs per shipment (median: 40; range: 1–700). Movements from farm to slaughterhouses averaged 29.7 pigs moved (median: 20, range: 1–400), while movements from farm to market were smaller, with an average number of pigs moved of 8.2 (median: 7; range: 0–80). Movements from market to a farm averaged 8.0 pigs moved (median: 5; range: 1–30), while the 2 movements from market to slaughterhouses had 4 pigs. The majority (83.0%) of movements from farm to market resulted in a record of zero pigs arriving. Excluding these zero arrival records, the average difference between number of pigs shipped and number of pigs arriving was <0.1 (median: 0; range: 0–40).

## Discussion

This study summarized North Macedonian pig population census data over the last 5 years, and provided one of the first descriptions of their live pig movement network. The number of reported pigs and pig farms has increased during 2016–2020. The network of movement of these pigs was weakly connected, with instability across years among those farms with infrequent movements. The top shippers and receivers of live pigs were more consistent. Movements to slaughterhouses predominated the network. Most movements occurred within 50 km. These data are expected to provide key insights for targeted, risk-based, and economically efficient mitigations for disease spread. Further, these data allow us to make comparisons between North Macedonia and other countries in the region, and throughout Europe, and support the development of regional training materials and intervention strategies.

While pig producing countries around the world are observing a consolidation of pig production into larger-scale, commercial farms (26, 29, 35, 50, 51), the census data in North Macedonia reported smallholder farms taking up an increasingly larger proportion of the industry. The low median number of pigs per farm is consistent with this predominance of backyard farms in North Macedonia. The higher number of pigs per farm in Vardar reflects the higher density of commercial farms in that region (6). The observed increase in smallholder farms may represent a true increase or reflect improved rates of discovery and inclusion in the national registry. The highest numbers of pigs and farms were reported in 2018 and 2020, both years in which there was a financial incentive provided for each farm reported. This suggests that rather than true growth in the number of smallholder farms, there is a proportion of these farms that has historically not been consistently captured in the pig census. Within the Polog region, a decrease in the number of farms and increase in the number of pigs was observed, suggesting that, at least in this region, North Macedonia's swine industry is following the trend toward consolidation. One of the benefits of consolidation into commercial production systems is the general increase in biosecurity standards of these farms. Within North Macedonia, the high density of smallholder farms in the Northeastern region bordering ASF-positive Serbia and Bulgaria, and Eastern region, as the location of North Macedonia's recent ASF introduction, is concerning. As an area with a high number of low biosecurity premises, consistent documentation of premises in these regions is critical to enabling risk-based awareness events and trainings, and targeted disease surveillance and mitigation efforts.

North Macedonia closed animal markets to pigs in August 2019 as part of their increased efforts to reduce the risk of ASF introduction. Though markets received about the same proportion of movements (even with partial 2019 data), no data on movements out of markets were reported during 2019. Markets predominantly remained closed during the COVID pandemic, briefly re-opening when restrictions were temporarily eased. With the recent ASF introduction, there is a ban on movement of swine except to slaughterhouses under controlled conditions. Given the historically poor traceability of pigs arriving at and sold from markets, the ongoing closure of these sites is recommended until the outbreak is controlled and record-keeping can be improved. Evaluation of movements by origin and destination type across time (Table 3), did show a reduction in the number of movements with an unknown destination, suggesting North Macedonia is doing a better job with movement records. However, the lack of reporting on arrival numbers, dates and sales, indicate ongoing efforts to enhance reporting are warranted. Movement data for the remainder of 2019 and 2020 was not available at the time of this analysis, therefore it is unclear how the network may adapt to the removal of market sites. The increased use of the slaughterhouse in the Southeastern region may indicate a shift from markets to slaughterhouses. This would be expected to reduce the risk of disease transmission *via* live pig movements, by increasing terminal movements. The closure of markets in 2019 and missing data during the peak months of November and December may explain the drop in betweenness observed in the 2019 network. Further investigation is needed to assess whether network connectivity dramatically increases with the surge of end of year movements, however the large proportion of movements to market that occur in November-December suggest this is the case. The drop in betweenness and increase in community numbers in 2019, suggest the most recent network is significantly less connected than that of previous years. With ASF control measures in place, the network may be permanently reshaped based on movement restrictions and tighter control of animal markets. In general, the North Macedonia live pig network is highly localized, though a few network communities span the country. Ultimately, this may have limited the spread of ASF before movement controls were implemented.

North Macedonia's live pig network demonstrated a seasonality that is consistent with other European countries, and that aligns with the Easter and Christmas holidays (29, 52, 53). Previously reported survey data was consistent with our network observations, identifying peak periods for slaughter of piglets in April to May and November to January, and for fattened pigs at the end of the year (6). Backyard and family farms also demonstrated a seasonal peak in the buying of new pigs, from March to May, contributing to additional movements during this time of year. While increased movements to slaughterhouses are not expected to contribute to a large risk of disease spread (assuming good waste management practices and no access of free-ranging pigs or wild boar to infected offal), peaks in movements associated with purchasing of new animals may contribute to a higher risk during the spring season (29). Implementation of pre-movement isolation periods, i.e., stopping movements into and out of a premises, and maintaining very high biosecurity, for a set period of time (e.g., one ASF incubation period) before shipping, during these seasonal peaks may increase the chance of observing a sick pig before shipment, and therefore decrease the risk of spreading disease.

As observed in many animal-production networks, large commercial farms and slaughterhouses acted as consistent shippers and receivers of live pigs in North Macedonia's network (26, 27, 29). Targeting those farms that ship most frequently, to the most other farms, and to non-slaughter destinations, for increased surveillance and training on the recognition of clinical signs and improvement of biosecurity, is expected to decrease the dissemination of disease in this network. While commercial and slaughterhouses premises may provide consistency to the network, the high turnover of UINs by farms in North Macedonia suggests that smallholder farms may not maintain pigs year to year. The shift in municipalities shipping low numbers of pigs between 2017 and 2019 may reflect the instability in this group of producers, or inconsistent documentation of these farms and their movements. Improved implementation of UIN assignments, and maintaining consistency in these assignments, across years is expected to improve traceability and thus disease response efficiency.

North Macedonia's weakly connected live pig movement network, with low diameter and average path length, and right-skewed indegree and outdegree, is consistent with networks described for other backyard predominant countries including Georgia (54), Bulgaria, Extremadura (Spain) and Côtes-d'Armor (France) (25, 55). EU member countries demonstrate much larger networks, with more community structure, and are additionally more likely to be impacted by international trade and movement of pigs (24, 27, 56). North Macedonia's weak connectivity may provide an advantage in limiting disease spread if network vulnerabilities are appropriately targeted during a disease outbreak.

Consistent with previously reported trade information, none of the reported movement data indicated export or trade of live pigs with EU member states or other countries (56). Bosnia and Herzegovina, Kosovo, Montenegro and Serbia historically reported exporting no live pigs; only Serbia reported export of pig products (56). The disconnected nature of North Macedonia's live pig network, especially in more recent years, and the lack of international trade, suggest the legal movement of live pigs is likely a low risk for disease spread in this region. This evaluation and the resultant risk-based recommendations for targeted interventions are limited to those supported by the live pig movement network. Additional information on the movement of pork products, vehicles, fomites, farm workers, veterinarians, and the illegal movement of live pigs and pork products, together with wild boar-related factors, is needed to make a better assessment of disease risk in the country. Indeed, these other factors are often seen as more important in the epidemiology of ASF than the movement of live animals.

This study has provided a foundation of information about the documentation and traceability of pigs in North Macedonia, and evidence to support ongoing improvement in this system. A better understanding of the live pig movement network has provided sites for targeted training and mitigation efforts, providing cost-effective, risk-based approaches to reduce the risk of disease introduction and spread. Future efforts will need to explore additional data sources, risk pathways, and modeling efforts to understand how this information may impact the spread of transboundary animal diseases, such as ASF, within North Macedonia's pig sector. The instability of North Macedonia's live pig movement network suggests that annual updates should be performed to analyses and resulting recommendations.

## Data availability statement

The data analyzed in this study is subject to the following licenses/restrictions: The data that support the findings of this study may be available on reasonable request from the corresponding author BM-L. The data are not publicly available due to the inclusion of private producer information. Requests to access these datasets should be directed to BM-L, beamartinezlopez@ucdavis.edu.

## Author contributions

KO'H performed the initial draft preparation, data curation and validation, development of the R-code, and formal analysis under supervision of BM-L. BT organized the raw data and contributed to data validation. BM-L supervised the development and implementation and interpretation of the analytic approach. All authors contributed to the project conceptualization and critical and extensive review and editing of the submitted manuscript.

## Funding

Data analysis was supported by the Ecology and Evolution of Infectious Diseases Program, Grant No. 2019-67015-28981 from the USDA National Institute of Food and Agriculture, the UC Davis Graduate Group in Epidemiology, and by the Technical Cooperation Programme (TCP) project on African swine fever Preparedness in the Balkans (TCP/RER/3704) of the Food and Agriculture Organization (FAO).

## Conflict of interest

The authors declare that the research was conducted in the absence of any commercial or financial relationships that could be construed as a potential conflict of interest.

## Publisher's note

All claims expressed in this article are solely those of the authors and do not necessarily represent those of their affiliated organizations, or those of the publisher, the editors and the reviewers. Any product that may be evaluated in this article, or claim that may be made by its manufacturer, is not guaranteed or endorsed by the publisher.

## Author disclaimer

The views expressed in this paper are those of the author(s) and do not necessarily reflect the views or policies of FAO.
