# Supplementary material for: Network analysis of live pig movements in North Macedonia: Pathways for disease spread
Source: Front Vet Sci. 2022 Aug 9;9:922412. doi: 10.3389/fvets.2022.922412 (PMC9396142; doi:10.3389/fvets.2022.922412)
Supplement: Supplementary file 1 [file Data_Sheet_1.pdf]

**Supplemental:**

Supplemental Figure S1. Frequency of occurrence of a given unique identification number (UIN) in the North Macedonia pig census between 2016-2020.

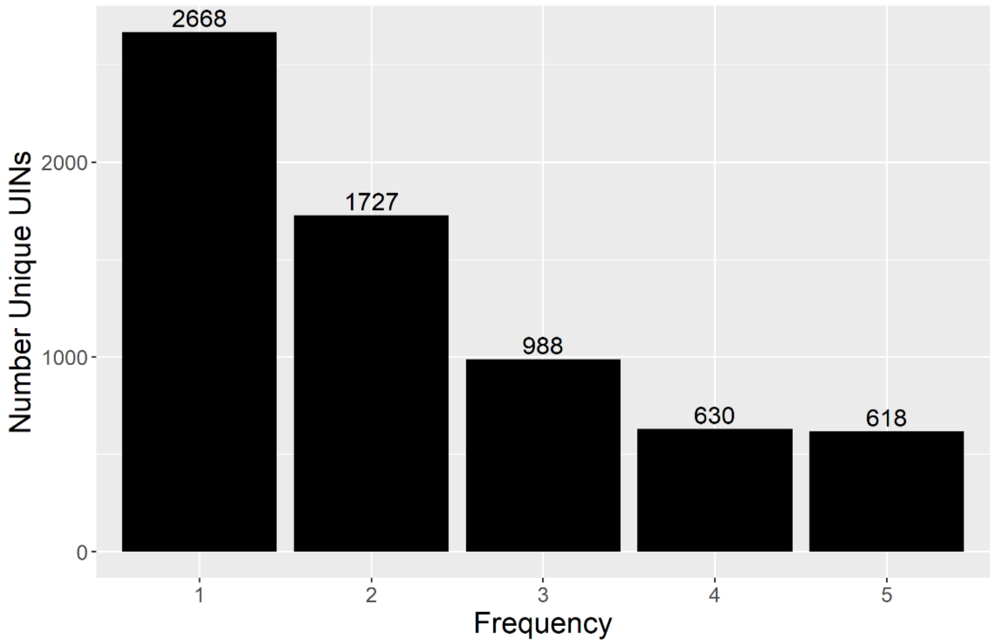

Supplemental Figure S2. North Macedonia live animal movement networks' degree distributions for a) 2017, b) 2018, and c) 2019, and power-law degree distributions on log-log scale for d) 2017, e) 2018, and f) 2019 where k is degree.

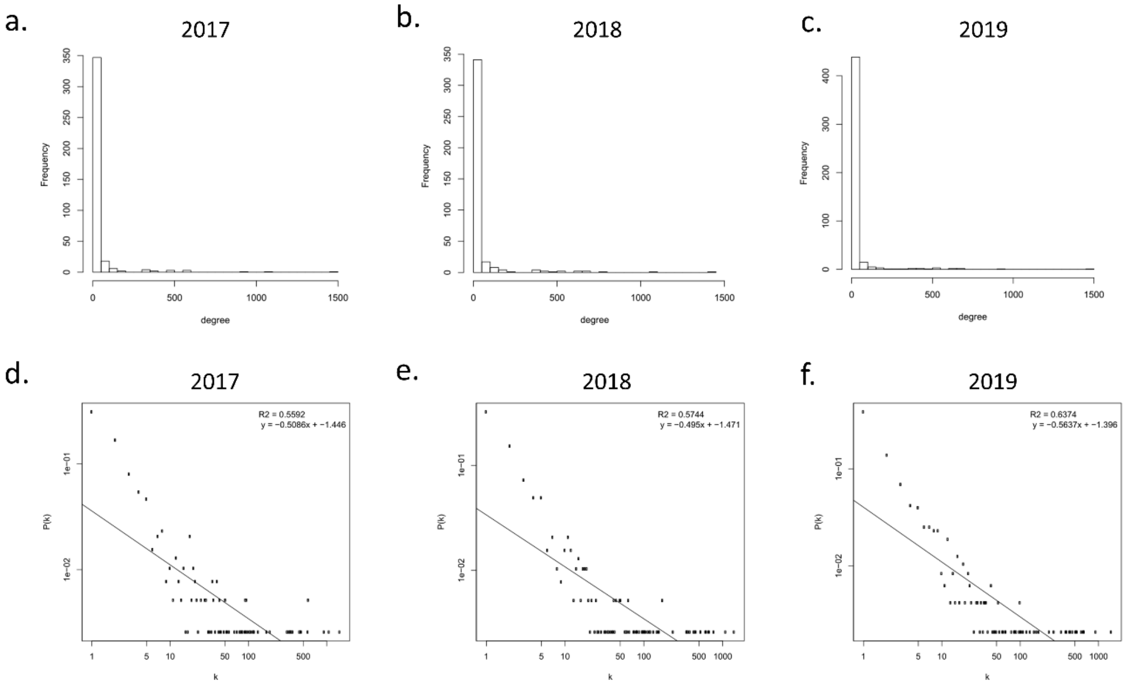

Supplemental Figure S3. Distance (km) of live swine shipments in North Macedonia for a) 2017-2019, b) 2017, c) 2018, and d) 2019.

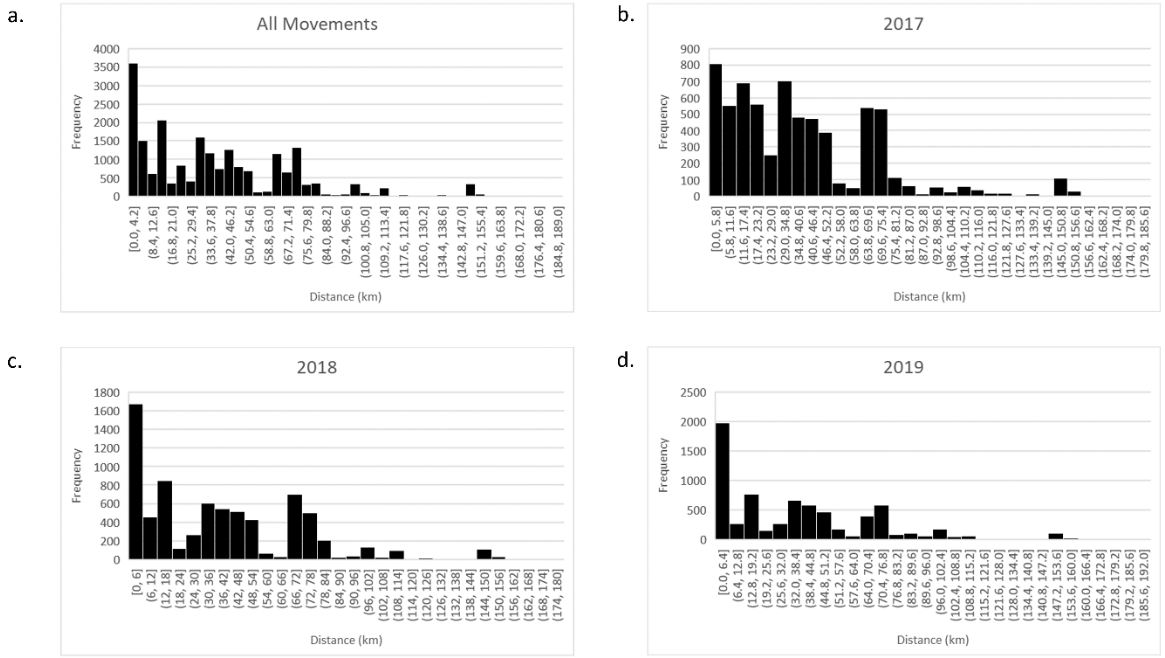

**Supplemental Tables:**

Supplemental Table S1. North Macedonia pig demographics based on census data for 2016-2020. Summary data by total number of pigs, piglets, fattening pigs, gilts, sows and boar per farm. Farms reporting zero total animals were excluded.

| | | 2016 | 2017 | 2018 | 2019 | 2020 |
|---|---|---|---|---|---|---|
| **Total** | **Avg** | 52 | 47 | 42 | 58 | 46 |
| | **Median** | 5 | 4 | 3 | 4 | 3 |
| | **Minimum** | 0 | 1 | 1 | 1 | 1 |
| | **Maximum** | 18576 | 19837 | 21747 | 22459 | 21159 |
| **Piglets** | **Avg** | | | 17 | 25 | 18 |
| | **Median** | | | 0 | 0 | 0 |
| | **Minimum** | | | 0 | 0 | 0 |
| | **Maximum** | | | 11058 | 10996 | 9955 |
| **Fattening** | **Avg** | | | 19 | 25 | 22 |
| | **Median** | | | 0 | 0 | 1 |
| | **Minimum** | | | 0 | 0 | 0 |
| | **Maximum** | | | 8488 | 9273 | 8930 |
| **Gilts** | **Avg** | | | 1 | 1 | 1 |
| | **Median** | | | 0 | 0 | 0 |
| | **Minimum** | | | 0 | 0 | 0 |
| | **Maximum** | | | 680 | 665 | 592 |
| **Sows** | **Avg** | | | 4 | 7 | 5 |
| | **Median** | | | 1 | 2 | 1 |
| | **Minimum** | | | 0 | 0 | 0 |
| | **Maximum** | | | 1498 | 1495 | 1657 |
| **Boars** | **Avg** | | | 0 | 0 | 0 |
| | **Median** | | | 0 | 0 | 0 |
| | **Minimum** | | | 0 | 0 | 0 |
| | **Maximum** | | | 28 | 30 | 25 |

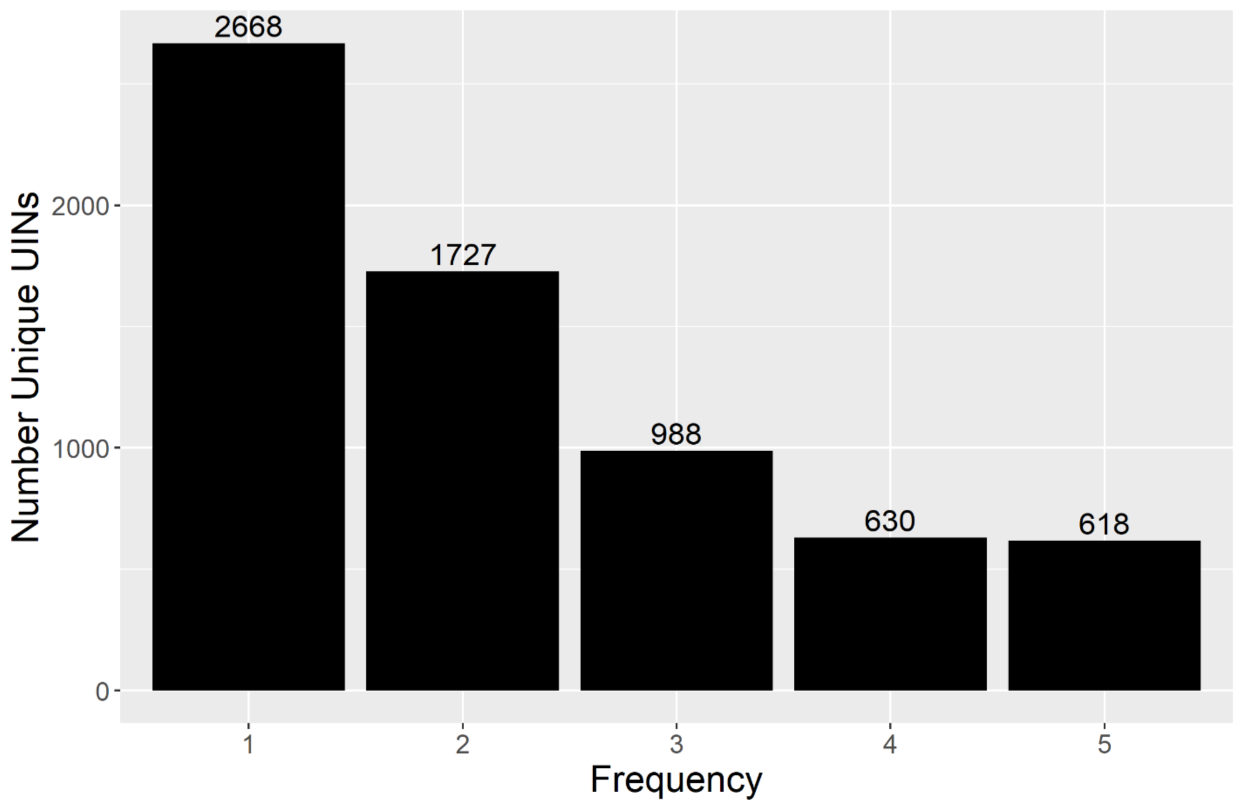

2668
1727
988
630
618
Number Unique UINs
2000
1000
0
1
2
3
4
5
Frequency
